# To Leave or Not to Leave? A Multi-Sample Study on Individual, Job-Related, and Organizational Antecedents of Employability and Retirement Intentions

**DOI:** 10.3389/fpsyg.2019.02057

**Published:** 2019-09-10

**Authors:** Pascale M. Le Blanc, Maria C. W. Peeters, Beatrice I. J. M. Van der Heijden, Llewellyn E. van Zyl

**Affiliations:** ^1^Human Performance Management Group, Eindhoven University of Technology, Eindhoven, Netherlands; ^2^Department of Social, Health and Organizational Psychology, Utrecht University, Utrecht, Netherlands; ^3^Institute for Management Research, Radboud University, Nijmegen, Netherlands; ^4^School of Management, Open University of the Netherlands, Heerlen, Netherlands; ^5^Department of Marketing, Innovation and Organisation, Ghent University, Ghent, Belgium; ^6^Hubei Business School, Hubei University, Wuhan, China; ^7^Kingston Business School, Kingston University, London, United Kingdom; ^8^Optentia Research Focus Area, North-West University, Vanderbijlpark, South Africa

**Keywords:** older workers, employability, retirement intentions, training and education, learning value of the job, organizational career management practices, health, financial situation

## Abstract

In view of the aging and dejuvenation of the working population and the expected shortages in employees’ skills in the future, it is of utmost importance to focus on older workers’ employability in order to prolong their working life until, or even beyond, their official retirement age. The primary aim of the current study was to examine the relationship between older workers’ employability (self-)perceptions and their intention to continue working until their official retirement age. In addition, we studied the role of potential antecedents of their perceived employability at three different levels: training and education in current expertise area as well as in an adjacent expertise area (individual level factor), learning value of the job (job level factor), and organizational career management practices (organizational level factor). Data were collected by means of e-questionnaires that were distributed among two groups of Dutch older (45-plus) white collar workers. The samples consisted of 223 employees of an insurance company, and 325 university workers, respectively. Our research model was tested separately in each sample using Structural Equation Modeling. We controlled for effects of respondents’ (self-)perceived health and (self-)perceived financial situation. Similar results were found for both samples. First, the relationship of perceived employability with the intention to continue working until one’s retirement age was positive, whereas the relationship between a perceived good financial situation with the intention to continue working until one’s retirement age was negative. Secondly, as regards the potential antecedents, results showed that the learning value of the job was positively related to perceived employability. In addition, an employee’s perception of good health is a relevant correlate of perceived employability. So, whereas perceived employability contributes to the intention to continue working until one’s retirement age, a good financial situation is a push factor to retire early. In order to promote the labor participation of older workers, this study indicates that organizations should focus on the learning possibilities that are inherent to one’s job rather than on providing additional training or career management. Further research is needed to test the generalizability of our results to other samples.

## Introduction

In the past decades, life expectancy at age 65 has greatly increased across OECD countries (Organization for Economic Co-operation, and Development [OECD], 2018). As the official retirement age in the Netherlands (where this study was performed) is linked to life expectancy, the average retirement age has risen too, from 62.7 years in 2011 to 64.8 years in 2017. Still, almost 40% of the workers is resigning from work before their official retirement age (CBS, 2018). In view of the global aging and dejuvenation of the working population (Bal et al., 2015), and the expected shortages in employees’ skills in the future (World Economic Forum, 2018), it is of utmost importance to focus on older workers’ employability in order to prolong their working life until their official retirement age. Moreover, due to the aging of the baby boom generation and the associated costs for retirement of this large cadre of people, it is also important to prevent early retirement intentions of this cohort (Beehr and Bennet, 2007). Due to the aforementioned issues, the official retirement age in the Netherlands is gradually being increased to 67.3 years in 2022.

Nevertheless, until recently, late-career issues have been largely ignored in academic research (Van der Heijden and De Vos, 2015; Veth et al., 2018). The aim of the current paper is to contribute to filling this gap in the literature, by focusing on the factors that are associated with the retirement intentions of individuals in their late career. In particular, given the fact that people will have to work longer in the (near) future, empirical research that sheds more light on factors that can help them to remain motivated throughout their working life (Zappala et al., 2008) and that safeguard and further enhance their employability (Van der Heijde and Van der Heijden, 2006; Van der Heijden et al., 2018) is badly needed. This especially applies to the group of older (45-plus) workers. Nielsen (1999) showed that, compared to their younger colleagues, older workers in Denmark are more worried about obtaining adequate employment based on their age and competences, i.e., about their (future) employability (see also Dordoni and Argentero, 2015; Neumark et al., 2019). Results of a study in the Netherlands by Van der Heijden (2002) revealed that managers are generally of opinion that workers’ employability decreases with age. Thus, employability is a key issue for elderly workers, and sound policies and practices are needed that should be aimed at sustaining older workers’ ability and motivation to work healthily and motivated until their actual retirement age (Van der Heijden et al., 2008).

Until recently, scholars mainly focused on workers’ reasons for retiring early, both on the individual level (e.g., poor health, pension benefits, and marital/family status; e.g., Beehr and Bennet, 2007) and on the job level (i.e., specific demands and/or resources; e.g., Schreurs et al., 2011) (see also Topa et al., 2018, for an exemplary meta-analysis). We take the opposite point of view by focusing on why people want to work until their official retirement age instead of retiring early. In addition to studying the effects of factors at the *individual* (training/education) and at the *job* level (learning value of the job) respectively, we decided to include a factor at the *organizational* level (organizational career management practices) as well, as prior research has shown that organizational HR practices may influence employee attitudinal and behavioral outcomes (Guest, 1999; Gratton and Truss, 2003; Akkermans et al., 2019).

Moreover, there have been repeated calls for an investigation of factors that may intervene in the relationship between HR practices and individual and/or organizational outcomes (Ferris et al., 1998; Wright et al., 2003; Boselie et al., 2005; Ostroff and Bowen, 2016; Veth et al., 2018). Hence, gaining empirical insight into how (i.e., through which mediators) specific HR practices may impact these outcomes - the so-called ‘black box’ of HRM (Ramsay et al., 2000; Becker and Huselid, 2006) – is considered as one of the key issues in the HRM research field (Veth et al., 2018). In the present study, we draw on the Job Demands-Resources (JD-R) model (Demerouti et al., 2001) by studying the relationship of specific resources at three different levels (individual, job, and organization) with employees’ self-perceived employability (cf. Van der Heijden, 2005), and further with their retirement intentions.

To summarize, the aim of the current study was threefold: Firstly, to examine the relationship between older (45-plus) workers’ employability perceptions and their intention to continue working until their official retirement age. Secondly, to simultaneously study the role of potential antecedents of older workers’ perceived employability at three different levels: training and education in one’s current expertise area as well as in an adjacent expertise area (*individual level*), learning value of the job (*job level*), and organizational career management practices (*organizational level*). And thirdly, to probe the possibly mediating role of perceived employability in the relationship between these specific antecedent factors and older workers’ intention to continue working until their official retirement age. Our study makes the following contributions to the literature. Firstly, it shifts the focus from older workers’ reasons to retire early to older workers’ reasons to continue working until their official retirement age. Secondly, it aims to uncover the mechanism underlying the relationships between specific work-related factors at the individual, job, and organizational level with the intention to continue working until one’s official retirement age by proposing perceived employability as a possible mediator. In addition, it sheds light on the relative importance of the specific distinguished antecedent factors for older workers’ perceived employability. In the following paragraphs, the core constructs of our study and the hypothesized relationships between the variables in our research model will be discussed in more detail.

## Employability

Based on Van der Heijde and Van der Heijden (2006), we define employability as: “the continuous fulfilling, acquiring or creating of work through the optimal use of competences” (p. 453). An employable workforce is a happy, healthy and productive workforce (Van der Heijden, 2005) and is also of vital importance for organizations in order to enable them to adapt to a rapidly changing external environment (Baruch, 2009). In the past decades, the concept of employability has been defined on different levels (individual, organizational, and societal). As the focus of the current study was on individual employability perceptions of older workers, we used the thoroughly validated and psychometrically sound competence-based conceptualization of employability that was developed by Van der Heijde and Van der Heijden (2006). Four of the five subdimensions were included in the current study.

The first dimension is called occupational expertise. According to DeFillippi and Arthur (1996), people with a high amount of occupational expertise enjoy more benefits in terms of being offered more inter-firm career opportunities. As such, occupational expertise is a significant human capital factor that is vital for both individual employees and for their working organization. Obviously, in times of downsizing or restructuring, workers who are most likely to become redundant are the ones whose occupational expertise is lacking, obsolete, or outdated (Van der Heijden and De Vos, 2015).

The second and third dimensions of the competence-based employability construct concern adapting to changes and developments at both a job-content level and at other levels, such as the career as a whole, that are relevant in order to survive at today’s labor markets (Fugate et al., 2004; Van der Heijden et al., 2009b; Forrier et al., 2015). In particular, in the competence-based operationalization of employability that we used in this study, two different types of adaptation are distinguished, the first one being a self-initiating proactive variant that is referred to as anticipation and optimization, and the second one being a more passive, reactive variant entitled personal flexibility. Increasingly, employees have to enact their jobs and their professional life themselves (Weick, 1996), and have to take responsibility for individual agency (Van der Heijden and De Vos, 2015; De Vos et al., 2018), owing to the complexity of work and difficulty of employers to predict future knowledge and skills needs.

The fourth subdimension of employability that was incorporated in this empirical work comprises *corporate sense* which refers to the requisite social competence of individual workers and is defined as the “participation and performance in different work groups, and involves sharing responsibilities, knowledge, experiences, feelings, credits, failures, goals, etc.” (Van der Heijden, 2005, p. 26). All in all, self-perceived employability forms the basis for optimal employee functioning (Vanhercke et al., 2014), and sustainable careers (De Vos et al., 2018).

## Employability and the Intention to Continue Working Until One’s Official Retirement Age

The results of a study by Prothero and Beach (1984) substantiate the use of retirement intentions as a proxy for actual retirement behaviors. Analogously, according to Ajzen’s (1991) theory of planned behavior, intention to retire is the most proximal precursor of actual retirement behavior. So for both theoretical and practical reasons, this empirical study builds on earlier work that measured retirement intentions and is based on the notion that these are viable sources of information about retirement decisions (see also Ajzen, 2015).

From an individual, organizational, and social perspective, the logical question is why people consider and/or decide to retire. Important factors are labor market conditions, social security policies, and personal and social variables (Beehr, 1986; Reeuwijk et al., 2013). According to Van der Heijden (2005), workers are able to acquire and preserve specific competences that render them control over their own career in different organizations and fields of work. Moreover, the existing and newly developed competences contribute to their self-perceptions of employability (Camerino et al., 2008). We argue that positive self-perceptions of employability might extend people’s working lives (see also Gendron, 2011; Armstrong-Stassen and Stassen, 2013). As such, employability is considered to be a personal career resource (cf. De Coen et al., 2015) that is expected to influence employees’ retirement intentions (Wang and Shultz, 2010; Feldman and Beehr, 2011; see also Dordoni et al., 2017). Therefore, we formulated the following hypothesis:

Hypothesis 1: Older workers’ perceived employability is positively related to their intention to continue working until their official retirement age.

## Antecedents of Employability

According to Clarke and Patrickson (2008), employability management should be a shared responsibility of both employer and employee. Therefore, our hypothesized model builds upon an integrative approach of employability-enhancing activities, involving both the organization and the individual worker (Sandberg, 2000; Van der Heijde and Van der Heijden, 2006). We propose that both individuals’ participation in employability development initiatives and organizational career management practices are important for competency development (De Vos et al., 2011). In particular, we incorporate individual (training in current area of expertise and training in adjacent area of expertise), job-related (learning value of the job) and organizational-level (organizational career management practices) factors as possible antecedents in our research model. Earlier work already indicated the importance of formal and informal learning for employability (Van der Heijden et al., 2009a, b, 2016). As regards the learning value of the job, Van der Heijden et al. (2016) reported that this was an important antecedent of employability. In a similar vein, Van der Heijden and Bakker (2011) also found that the learning value of the job was an important predictor of employability, through its impact on work-related flow. As regards the organizational factor that we included in our research, that is organizational career management practices, we build upon the earlier work by Schneider et al. (1996), who posited that organizational support for competency development of their workers may not only enhance an individual’s domain-specific expertise, but also more general perceptions of employability (see also Nauta et al., 2009; De Vos et al., 2011). Below, each of these factors will be explicated in more detail.

### Individual Level: Training and Education

At the individual level, one’s employability can be enhanced by investing in human capital, i.e., one’s knowledge and skills (Forrier et al., 2015). According to human capital theory (Becker, 1993), participation in training and education enhances an individual’s employability (Berntson et al., 2006). Groot and Maassen van den Brink (2000) indeed showed that formal work-related training increases employability. Specifically, Van Vuuren et al. (2011) found that Life Long Learning is related to an increase in employability among 45-plus workers. In a similar vein, Dymock et al. (2012) performed a study among 250 older white collar workers and concluded that extending the working lives of these valued workers might be realized by providing them with ongoing education and training, in order to sustain their capacities and interest in contributing to their work and workplaces. This outcome is in line with the results of earlier studies that demonstrated that training and education related to the development of more general as well as function-specific skills contribute to increasing one’s employability (Forrier and Sels, 2003; Berntson et al., 2006; Van der Klink et al., 2014). Finally, the results of a European study by De Preter et al. (2013) found that participation in a training course helped to retain people in the labor market longer.

Hypothesis 2a: Training and education in current area of expertise is positively related to older workers’ perceived employability.
Hypothesis 2b: Training and education in adjacent area of expertise is positively related to older workers’ perceived employability.

### Job Level: Learning Value of the Job

We build upon earlier work by Van Dam et al. (2009) who posited that challenging and rewarding work aspects that are predictive for a worker’s level of employability, are positively related to the intention to continue working (longer), and incorporated the learning value of one’s job as an important antecedent in our study. The concept ‘learning value of the job’ was coined by Boerlijst et al. (1993) and refers to the extent to which workers believe that their knowledge and skills are enhanced through performing their job. Van der Heijden (2001) found that this learning value of the job is an important predictor of employability. Analogously, the earlier mentioned study by Groot and Maassen van den Brink (2000) showed that employees who occupy a complex function are more often deployed in other functions within their own organization, i.e., are more employable. More specifically, in their mediation model for employability enhancement, Van der Heijden and Bakker (2011) demonstrated that functions that enable employees to enlarge their competences lead to a higher level of work-related flow, which in turn contributed to their employability.

Hypothesis 3: The learning value of the job is positively related to older workers’ perceived employability.

### Organizational Level: Organizational Career Management Practices

Career management essentially comes down to a matching process of individual and organizational needs (Baruch, 2004) by means of mentoring employees in filling in their career (Verbruggen et al., 2008). Sustainable career management addresses this matching process with a long-term view in mind, by focusing on ways to ensure workers’ employability (De Prins et al., 2015). Organizational career management practices refer to organizational activities that are aimed to plan and to manage the careers of its employees (Sturges et al., 2002). It includes a wide range of programs and interventions that focus on aligning individual and organizational career needs (De Vos et al., 2008). According to Forrier and Sels (2003), adequate organizational career management practices may enhance one’s employability by enabling employees to better formulate their career expectations, by increasing their knowledge of the labor market, and by identifying and addressing the right persons to network with. In addition, it may stimulate employees to undertake activities themselves to further protect and foster their employability (Verbruggen et al., 2008).

Hypothesis 4: Organizational career management practices are positively related to older workers’ perceived employability.

## Perceived Employability as a Mediator

In addition to the direct associations between the three antecedent factors and perceived employability, we also propose perceived employability as a mediator in the relationship between the three distinguished factors, on the one hand, and the intention to continue working until one’s retirement age, on the other hand. As regards the individual level, investing more time and effort in training and education will enhance employability in terms of occupational expertise and adaptability (Van der Heijden, 2005), thereby probably boosting the worker’s confidence regarding the ability to continue working at a satisfactorily level until his/her retirement age (see also De Coen et al., 2015). In turn, this is likely to positively affect his/her intention to continue working until his/her official retirement age (Newman, 2011).

As regards the job level, we already indicated that a job that offers opportunities for learning and development is related to higher levels of work-related flow and, consequently, to being more employable (Van der Heijden and Bakker, 2011). A lack of work engagement, which is conceptually related to flow, is also supposed to be one of the main reasons to resign (De Lange et al., 2008), and has been associated with the intention to retire early in previous scholarly work (Siegrist et al., 2006; Vodopivec and Dolenc, 2008; Von Bonsdorff et al., 2010; Schreurs et al., 2011). With respect to working longer, Kooij et al. (2010) stated that older workers should be offered intrinsically motivating jobs, i.e., managers should assure interesting jobs in which their older staff can accomplish worthwhile tasks in order to keep them motivated and thus employable. The results of longitudinal research by Van Solinge and Henkens (2011), among workers (*N* = 1600) from three Dutch private sector organizations and civil servants, point in the same direction, as they found that older workers with attractive jobs have a higher (planned) retirement age.

As regards the organizational level, Akkermans et al. (2019) already identified employability as an explanatory mechanism in the HR practices – outcome (i.e., organizational commitment) relationship. In a similar vein, De Coen et al. (2015) showed that self-awareness and adaptability negatively relate to older workers’ intention to retire via multiple paths, including enhancing self-directedness that, in turn, leads to a higher self-perceived employability. Therefore, it is important to sustain employees’ competencies throughout their career by means of investing in organizational career management practices (Verbruggen et al., 2008; Savickas et al., 2009).

Hypothesis 5: Perceived employability mediates the relationship between training and education in current expertise (H5a) and adjacent expertise (H5b), learning value of the job (H5c), organizational career management practices (H5d) and employees’ intention to continue working until their retirement age.

### Control Variables: Perceived Health and Perceived Financial Situation

Poor or declining physical health status may preclude an individual from continuing to work (Fisher et al., 2016), and the most frequently cited reason for early retirement is poor health (Garcia-Gomez, 2011; Van Rijn et al., 2014). Results of the Survey on Health and Ageing in Europe (SHARE) showed that poor self-reported health is a strong predictor of labor force exit even after controlling for factors that are predictive of poor health such as obesity, problem use of alcohol, low job control and effort-reward imbalance (Van den Berg et al., 2010). On the other hand, Fasbender et al. (2014) found that the better older workers’ experienced subjective health in 1996 was, the higher their engagement in post-retirement employment in 2008. Therefore, we decided to control for the effects of perceived health in our research model.

In addition, retirement timing is also affected by financial resources that are available for retirement. According to rational choice theory (Laitner and Sonnega, 2013), people will retire when they feel that their accumulated financial resources and the forecast of future economic conditions allow them to meet their consumption needs in retirement (Wang and Wanberg, 2017), meaning that most people wait to retire until they feel that they can afford to do so (e.g., Beehr et al., 2000; Mein et al., 2000). A longitudinal study of Dutch workers aged 59–63 indeed found that having the financial ability to stop working before age 65 predicted the transition to non-disability retirement before the official retirement age (De Wind et al., 2015). As regards the post-retirement phase, Fasbender et al. (2016) found that retirees who reported better economic status were less likely to engage in post-retirement employment. Therefore, we also controlled for the effects of perceived financial situation in our research model.

## Materials and Methods

### Research Approach

A multi-sample cross-sectional online survey-based research design was employed to investigate the relationships between the variables. This design provided a means through which to explore the relationships at a given time-point within two separate organizations. The multi-sample (multi-study) design aided in managing the constraints and criticisms associated with traditional single assessment cross-sectional designs (Field, 2016).

### Procedure and Participants

Data were collected in 2011 by means of e-questionnaires that were distributed among two different groups of Dutch white collar workers aged over 45 years. The first group consisted of 500 randomly selected employees of a Dutch insurance company, of whom 223 (48.2%) responded. The second group consisted of all 688 employees working at three different faculties of a Dutch university, of whom 325 (47.2%) responded.

Of the 223 employees of the insurance company (Sample 1), 44.8% was female, and their ages ranged from 45 to 63 years (*M* = 51.9; *SD* = 4.4). The majority was married or cohabiting and with (28.3%) or without (50.2%) children living at home. Almost twenty percent of the respondents were single and with (7.2%) or without (11.2%) children living at home. Their educational level varied from primary education (0.4%), secondary education (32.7%), lower vocational education (22.9%), higher vocational education (27.8%) to university education (16.1%). On average, they had been working 19.6 years (*SD* = 10.5) for their current organization. Of the 325 employees of the Dutch university (Sample 2), 48.9% was female, and their ages ranged from 45 to 64 years (*M* = 54.2; *SD* = 5.4). The majority was married or cohabiting and with (41.5%) or without (37.8%) children living at home. Almost twenty percent of the respondents were single and with (5.5%) or without (12.6%) children living at home. Their educational level varied from primary education (0.3%), secondary education (7.1%), lower vocational education (3.4%), higher vocational education (15.4%) to university education (73.8%). On average, they had been working 18.3 years (*SD* = 10.5) for their current organization. Except for educational level (χ^2^ = 124.30; *p* < 0.001), there were no significant differences in demographics between the two samples.

### Measures

Several self-report instruments were employed to assess the focal study variables and the control variables.

#### Focal Study Variables

*Training* and *education* was measured by means of two items (Van der Heijden, 2001), i.e., “*How many days did you spend on training and education in your current area of expertise?*” and “*How many days did you spend on training and education in an adjacent area of expertise?*” For each of these items, respondents had to indicate how many days they had spent on this type of training and education in the past year. Responses were scaled and standardized.

The *learning value of the job* was measured by means of a reliable and valid six-item scale (Van der Heijden, 2001; Van der Heijden and Bakker, 2011), e.g., “My job enables me to exceed my limits” that are answered on a six-point scale ranging from ‘strongly disagree’ (1) to ‘strongly agree’ (6).

*Organizational career management practices* was measured by means of an eight-item scale (De Vos et al., 2008) asking respondents to what extent their organization offers the following support in developing and planning one’s career: (1) workshops about career planning, (2) readers about career planning, (3) workshops on retirement-related topics, (4) handbooks containing information on career options within their organization, (5) tailor-made personal career trajectories regarding different options within their organization, (6) center for career counseling, (7) career counseling sessions with specialized counselors, and (8) career counseling sessions with HR managers. Items were answered on a five-point scale ranging from ‘to a limited extent’ (1) to ‘to a large extent’ (5).

*Perceived employability* was measured by means of four subscales of the psychometrically validated competence-based measure by Van der Heijde and Van der Heijden (2006). The first subscale is called *occupational expertise*, and consists of fifteen items, e.g., “*I consider myself competent to engage in in-depth specialist discussions in my job domain*” that are answered on a five-point scale ranging from ‘not at all’ (1) to ‘extremely’ (5). The second subscale is called *anticipation and optimization*, and consists of eight items, e.g., “*I take responsibility for maintaining my labor market value*” that are answered on a five-point scale ranging from ‘not at all’ (1) to ‘to a considerable extent’ (5). The third subscale is called *personal flexibility* and consists of 8 items, e.g., “*I adapt to developments in my organization*,” that are answered on a 5-point scale ranging from ‘very poorly’ (1) to ‘very well’ (5). The fourth subscale is called *corporate sense*, and consists of seven items, e.g., “*I am involved in achieving my organization’s/department’s mission*” that are answered on a five-point scale ranging from ‘not at all’ (1) to ‘to a considerable extent’ (5).

*Intention to work until one’s retirement age* was measured by means of a four-item scale (Van Dam et al., 2009). A sample item is: “*I will continue working until I am 65 years old*” (which was the official retirement age in the Netherlands at the time we collected our data). Items were answered on a five-point scale ranging from ‘completely disagree’ (1) to ‘completely agree’ (4).

#### Control Variables

The first control variable *perceived health* was measured by means of the four-item scale by Adams and Beehr (1989). A sample item is: “*My health is better than that of most other people of the same age.*” Items were answered on a 5-point scale ranging from ‘completely disagree’ (1) to ‘completely agree’ (5). The second control variable *perceived financial situation* was measured by means of one item (Zappala et al., 2008), i.e., “*Would your current financial situation enable you to retire before the official retirement age?*” that was answered on a 7-point scale ranging from ‘absolutely not’ (1) to ‘completely’ (7).

### Statistical Analyses

Data was sequentially analyzed with both SPSS v26 (IBM, 2019) and Mplus v 8.3 (Muthén and Muthén, 2019). A similar three phased analytical process was followed for both Study 1 and Study 2. First, the distribution of the data for each study was estimated through descriptive statistics (means, standard deviations, skewness, kurtosis) and relationships through Pearson product-moment correlation coefficients (*r* > 0.30: medium effect; *r* > 0.50: large effect; *p* < 0.01, Field, 2016). The upper-bound level of internal consistency for the instruments was assessed through point-estimate composite reliability (ρ > 0.70; Wang and Wang, 2012). Phi was used as a more robust indicator of the upper-bound level of internal consistency (Wang and Wang, 2012). Further, the presence of common method bias (CMB) was assessed through a series of common latent factor methods as proposed by Podsakoff et al. (2003) and Tehseen et al. (2017).

Second, a confirmatory factor analytical approach through structural equation modeling (SEM) was employed to sequentially compare competing measurement models in order to determine the best fitting model for the structural model in both studies. The robust mean-adjusted weighted least squares (WLSM) estimation method was employed (Muthén et al., 1997). The WLSM estimation method allows for the simultaneous processing of categorical and continuous variables (Asparouhov and Muthén, 2010; Wang and Wang, 2012) and provides stable, reliable approximations of model parameter estimates in smaller samples (Nussbeck et al., 2006; Kline, 2011; Savalei and Rhemtulla, 2013). Observed variables were treated as indicators for the latent factors. Model fit was determined through (1) absolute fit-, (2) approximate fit-, and (3) comparative fit indices (c.f. Table 1). Given that estimated models cannot directly be compared with the WLSM estimation method, the Satorra–Bentler chi-square difference test (Satorra and Bentler, 2010) method was employed to determine differences between measurement models.

**TABLE 1:** Fit indices: acceptable values and cut-off points.

**Fit indices**	**Acceptable values**
**Absolute fit indexes**	
Chi-Square	Lowest value in comparative measurement models Significant Chi-Square Difference (*p* > 0.001)
Standardized Root Mean Residual (SRMR)	SRMR < 0.08
**Approximate Fit Indexes**	
Steiger-Lind Root-Means-Square Error of Approximation (RMSEA)	RMSEA < 0.08 90% Confidence Interval < 0.08 but >0.01
**Incremental fit indices**	
Comparative Fit Index (CFI)	>0.90: Acceptable; >0.95: Excellent
Tucker-Lewis Index (TLI)	>0.90: Acceptable; >0.95: Excellent

*Adapted from Nussbeck et al. (2006), Kline (2011), and Savalei and Rhemtulla (2013).*

Third, based on the best-fitting measurement model, a structural model was estimated. Items were permitted to correlate freely, and no additional constraints were placed on the models. In both the measurement- and structural models, perceived health and perceived financial situation was controlled for by regressing such on perceived employability and intention to work until one’s retirement age. The results were reported in line with the best practice guidelines for SEM by Morrison et al. (2017).

Finally, the indirect effects of perceived employability on the relationship between the significantly loading exogenous and endogenous factors were assessed with the bias-corrected bootstrapping (BCB) method (Preacher et al., 2010). A 50,000 BCB, at the 95% confidence interval (CI) limit, was employed to generate confidence interval changes and standard errors for the indirect effect assessment. When the 95% CI range does not include zero, an indirect effect is present (Wang and Wang, 2012).

Control variables (perceived health and perceived financial situation) were added to both the measurement and structural models. These variables were regressed on the endogenous latent variables (perceived employability and intention to work until one’s retirement age) (Schjoedt and Bird, 2014). Should model fit statistics change, or the strength of the regression paths change when control variables are included, it acts as an indicator that these factors significantly influence the outcomes (Schjoedt and Bird, 2014; Field, 2016).

## Results

First, the general descriptive statistics, correlations and composite reliabilities will be reported for each study. Second, the competing measurement models for each study will be discussed. Third, the results of the structural models will be tabulated for each study. Finally, the indirect effect results will be presented for each study.

### Descriptive Statistics, Composite Reliabilities, Pearson Product-Moment Correlations and Common Method Bias (CMB)

The presence of CMB was assessed through a series of sequential and increasingly restrictive assessments for both studies. First, Harman’s single factor test was performed. No single component could be extracted from an unrotated exploratory factor analyses for either study. The common shared variance for the single factor was below 35% (Podsakoff et al., 2003) for both studies. Second, a single factor CFA was performed in Mplus for both samples, whereby all observed variables were estimated to directly load on a common latent variable. This test also failed to produce adequate model fit [Sample 1: χ^2^_(2344, *N* = 223)_ = 8273.89; CFI = 0.32; TLI = 0.30; RMSEA = 0.11; SRMR = 0.12; Sample 2: χ^2^_(2344, *N* = 325)_ = 11581.07; CFI = 0.29; TLI = 0.27; RMSEA = 0.10; SRMR = 0.12] and thus failed to produce a single factor (Tehseen et al., 2017). Finally, a common latent factor was constructed within each sample with regression lines (which are constrained to be equal) leading to each observed indicator within the measurement model. The common latent variable’s variance was constrained to be 1 (Podsakoff et al., 2003). This model was then compared to an unconstrained model for each sample. The results showed that the common variance explained by the common factor was low (Study 1: *R*^2^: 0.06; Study 2: *R*^2^: 0.08) and the paths between variables are similar to the model without the common factor. Resultantly, CMB is ruled out for both samples.

Further, the descriptive statistics, Pearson product-moment correlations and composite reliabilities are presented in Table 2. The results showed that (with the exclusion of the two training and education variables) all variables for both studies were normally distributed, with Skewness and Kurtosis being smaller than the suggested range of −1 and 1. Given the large reported range within both of the single item training and education variables, both were standardized with the STANDARDIZE command in Mplus. Here the mean score for both factors was subtracted and then divided by the standard deviation. Further, all the scales showed high levels of upper-bound internal consistency with Rho’s phi being higher than 0.70 for each factor of each study (Wang and Wang, 2012).

**TABLE 2:** Descriptive statistics, composite reliabilities and Pearson Correlations for Study 1 and Study 2.

**No.**	** Factor**	**Study 1**					**Study 2**																
		**μ**	**σ**	***SK***	***Rku***	**ρ**	**μ**	**σ**	***SK***	***Rku***	**ρ**	**1**	**2**	**3**	**4**	**5**	**6**	**7**	**8**	**9**	**10**	**11**	**12**
1	Training (Specialist)	6,57	10,95	4,28	22,30	–	12,89	53,32	5,38	28,72	–	–	0,79	0,08	0,05	0,05	0,11	0,11	−0,03	−0,02	0,11^∗^	0,03	0,06
2	Training (New Domain)	2,78	7,35	6,30	48,65	–	7,88	40,15	7,65	61,99	–	0,36^∗^	–	0,05	0,06	0,02	0,06	0,12^∗^	−0,05	−0,06	0,10	0,04	0,02
3	Learning Value of the Job	4,32	0,86	−0,84	0.99	0,85	4,40	0,87	−0,69	0,70	0,81	0,26^∗^	0,04	–	0,02	0,36	0,23^∗^	0,34^∗^	0,23^∗^	0,31^∗^	0,25^∗^	0,08	0,07
4	Organizational Career Management Practices	2,45	0,90	−0,03	−0,85	0,94	2,02	0,83	0,36	−0,96	0,94	−0,01	−0,04	0,12	–	−0,02	−0,10	0,03	−0,04	0,01	−0,06	−0,09	0,07
5	Perceived Employability (Overall)	4,34	0,51	−0,13	0,13	0,79	4,44	0,54	−0,15	−0,36	0,81	0,17^∗^	0,08	0,34^∗^	0,08	–	0,73^∗^	0,81^∗^	0,73^∗^	0,83	0,23^∗^	0,16^∗^	0,02
6	Occupational Expertise	4,89	0,49	−0,23	0,30	0,92	4,95	0,52	−0,58	0,68	0,90	0,09	−0,01	0,13	−0,02	0,74^∗^	–	0,49^∗^	0,46^∗^	0,48	0,23^∗^	0,17^∗^	−0,03
7	Anticipation and Optimization	3,99	0,74	−0,03	−0,20	0,87	4,12	0,81	−0,12	−0,17	0,86	0,23^∗^	0,14^∗^	0,36^∗^	0,07	0,82^∗^	0,47^∗^	–	0,43^∗^	0,53	0,27^∗^	0,15^∗^	−0,06
8	Personal Flexibility	3,82	0,45	−0,35	0,08	0,78	3,76	0,52	−0,13	0,16	0,82	0,06	0,04	0,31^∗^	0,07	0,77^∗^	0,51^∗^	0,49^∗^	–	0,49	0,11^∗^	0,18^∗^	0,11
9	Corporate Sense	4,13	0,79	−0,16	−0,28	0,86	4,39	0,83	−0,36	−0,33	0,87	0,12	0,04	0,26^∗^	0,11	0,86^∗^	0,52^∗^	0,58^∗^	0,56^∗^	–	0,12^∗^	0,04	0,05
10	Intention to Work Until Retirement	3,35	1,05	−0,19	−0,72	0,92	3,94	1,07	−0,92	0,04	0,95	0,11	0,09	0,07	0,08	0,30^∗^	0,22^∗^	0,27^∗^	0,31^∗^	0,18	–	0,10	−0,15^∗^
11	Health	3,81	0,84	−0,94	0,71	0,91	3,85	0,85	−0,96	0,97	0,95	−0,01	0,06	0,15^∗^	0,00	0,36^∗^	0,30^∗^	0,31^∗^	0,30^∗^	0,26	0,19^∗^	−	0,04
12	Financial Situation	3,53	1,64	0,20	−0,65	–	3,63	1,99	0,11	−1,18	–	0,10	−0,01	0,12	−0,01	0,10	0,00	0,07	0,06	0,17	−0,19^∗^	0,07	–

*^∗^*p* < 0.01; Study 1’s correlations below the diagonal; Study 2’s correlations above the diagonal; μ, mean; σ, standard deviation; SK, skewness; Rku, kurtosis; ρ, phi (composite reliability).*

The Pearson Correlations showed statistically significant relationships amongst most of the variables within both samples (*p* < 0.01), however, the anticipated relationship between organizational career management practices and perceived employability was non-significant. Further, training and education in current expertise area was found to positively relate to learning value of the job within Study 1. Further perceived health related positively to perceived employability, and perceived financial situation negatively to intention to work until one’s retirement age in both samples.

### Estimating and Comparing Competing Measurement Models

Next, a competing measurement modeling strategy, employing a confirmatory factor analytical (CFA) approach, was used to determine the best fitting model for each study. Here observed variables were classified as continuous indicators for latent variables. Item 4 on the ‘learning value of the job’ scale (“*My job enables me to push the boundaries of my capabilities*”) was removed from subsequent analysis due to a non-significant factor loading on the *a priori* construct within both samples (*p* > 0.01).

Four competing theoretical permutations of the various instruments were constructed and systematically compared. All items were directly loaded onto their *a priori* theoretical constructs and independent (exogenous) variables were permitted to freely correlate. The following models were assessed:

• Model 1. Training and education (current expertise), training and education (adjacent expertise) and perceived financial situation were used as single observed exogenous indicators. Learning value of the job, organizational career management practices, intention to work until one’s retirement age and perceived health were specified as single factor latent variables. Perceived employability was specified as a hierarchical second order latent variable, comprising out of four first order latent variables (occupational expertise, anticipation and optimization, personal flexibility, and corporate sense).
• Model 2. Similar to Model 1, but perceived employability was specified as a one factor latent variable.
• Model 3. Similar to Model 2, but training and education (current expertise) and training and education (adjacent expertise) were used as indicators for a first order latent variable called ‘professional development.’
• Model 4. Professional development (training and education in current- and training and education in adjacent expertise), learning value of the job, organizational career management practices, intention to continue working until one’s retirement age, and perceived health were specified as first order latent variables. Perceived financial situation was again kept as a single order observed variable. Perceived employability was specified as a hierarchical second order latent variable, comprising out of four first order latent variables (occupational expertise, anticipation and optimization, personal flexibility, and corporate sense).

The results of the competing measurement models for both Studies are shown in Table 3. To compare and contrast the competing measurement models, the Satorra–Bentler scaled chi-square correction method (Satorra and Bentler, 2010) was employed to estimate the differences in the χ^2^ (*c.f.* Table 4).

**TABLE 3:** Measurement model fit statistics for Study 1 and Study 2.

**Model**	**χ^2^**	***df***	***RMSEA***		***SRMR***	***CFI***	***TLI***
**Study 1 (*N = 223*)**							
Model 1	3846.09	1816	0.07	[0.068,0.074]	0.07	0.93	0.93
Model 2	5241.31	1820	0.09	[0.089,0.095]	0.09	0.88	0.87
Model 3	5128.87	1817	0.09	[0.087,0.093]	0.09	0.88	0.87
Model 4	4828.91	1817	0.09	[0.083,0.091]	0.08	0.89	0.88
**Study 2 (*N = 325*)**							
Model 1	5078.68	1816	0.07	[0.072,0.077]	0.07	0.95	0.95
Model 2	6943.39	1820	0.09	[0.091,0.095]	0.08	0.92	0.92
Model 3	7015.66	1817	0.09	[0.092,0.096]	0.08	0.92	0.91
Model 4	6322.97	1818	0.09	[0.085,0.090]	0.08	0.93	0.92

**TABLE 4:** Satorra–Bentler scaled chi square differences.

	**Δ Satorra–Bentler Scaled χ^2^**	**Difference Test Scaling Correction**	**Δ*df***	***p*-value**
**Study 1**				
Model 1 vs. 2	–1513.95	–0.53	4	*p* < 0.01
Model 1 vs. 3	–590.96	–1.24	1	*p* < 0.01
Model 1 vs. 4	–309.55	–1.79	2	*p* < 0.01
Model 2 vs. 3	–61.87	1.12	–3	*p* < 0.01
Model 2 vs. 4	–87.60	2.85	–2	*p* < 0.01
Model 3 vs. 4	–77.18	–2.33	–1	*p* < 0.01
**Study 2**				
Model 1 vs. 2	–803.18	–1.43	4	*p* < 0.01
Model 1 vs. 3	–994.93	–2.68	1	*p* < 0.01
Model 1 vs. 4	–83.66	–8.30	2	*p* < 0.01
Model 2 vs. 3	–2.13	5.39	–3	*p* < 0.01
Model 2 vs. 4	–46.14	9.52	–2	*p* < 0.01
Model 3 vs. 4	–149.82	–2.85	–1	*p* < 0.01

For *Study 1*, the results showed (whilst controlling for perceived health and perceived financial situation) that Model 1 fitted the data significantly better than the other models [χ^2^_(1816, *N* = 223)_ = 3486.09; CFI = 0.93; TLI = 0.93; RMSEA = 0.07; SRMR = 0.07]. Table 4 shows the Satorra and Bentler (2010) scaled χ^2^ differences (S-B χ^2^) between the competing measurement models. Here, the results showed a large comparative discrepancy between Model 1 vs. 2/3/4, Model 2 vs. 3/4 and Model 3 vs. 4 in the S-B χ^2^. Through the changes in S-B χ2 between these models, further evidence for support of best fit for Model 1 is presented. Model 1 showed comparatively lower S-B χ^2^.

Similar results were found for *Study 2*. Here, the results showed (whilst controlling for perceived health and perceived financial situation) that Model 1 also fitted the data significantly better than the other models [χ^2^_(1816, *N* = 223)_ = 5078.68; CFI = 0.95; TLI = 0.95; RMSEA = 0.07; SRMR = 0.07]. Again, significant comparative discrepancies between Model 1 vs. 2/3/4, Model 2 vs. 3/4 and Model 3 vs. 4 in the S-B χ^2^ were reported. Model 1 showed comparatively lower S-B χ^2^ than the other models. Therefore, Model 1 was retained in both studies for conversion to the structural model.

### Assessing the Structural Models

Next, the most parsimonious and best fitting measurement model (Model 1) was used to construct a structural path model for both studies. Further, in order to ensure the robustness of the models, the models with and without the control variables were systematically computed, compared and contrasted.

#### Structural Model for Study 1

**FIGURE 1:**
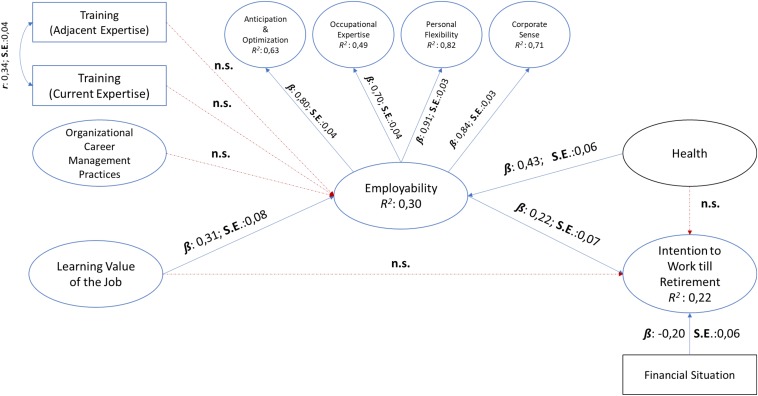
Structural model for Study 1 with control variables.

For Study 1, the structural model for the best-fitting measurement model (Model 1) showed acceptable fit *with* the control variables [*c.f.* Figure 1: χ^2^_(1809, *N* = 223)_ = 3855.00, *p* > 0.001; TLI = 0.93; CFI = 0.92; RMSEA = 0.07 [CI 95%:0.068,0.074]; SRMR = 0.07].

For the model with the control variables, the results showed that training and education (current expertise), training and education (adjacent expertise) and organizational career management practices did not produce statistically significant paths to perceived employability (*p* > 0.01). Further, learning value of the job related positively to perceived employability and declared 30% of the variance therein (β: 0.31; *SE*: 0.08; *p* < 0.01). Further, perceived employability statistically significantly predicted 22% of the variance within intention to work until one’s retirement age (β: 0.34; *SE*:0.08; *p* < 0.01). Within this sample, perceived health influenced perceptions of employability (β: 0.43; *SE*:0.06; *p* < 0.01) but not intentions to work until retirement (*p* > 0.01). Similarly, the perceived financial situation of individuals negatively influenced their intentions to work until retirement (β: −0.20; *SE*: 0.06; *p* < 0.01). Therefore, *Hypothesis 1*, which stated that perceived employability is positively related to intention to continue working until one’s retirement age, and *Hypothesis 3*, which stated that the learning value of the job is positively related to perceived employability, were *accepted* for Study 1. However, *Hypotheses 2a and 2b*, training and education in current area of expertise (H2a) and in adjacent area of expertise (H2b) is positively related to workers’ perceived employability, and *Hypothesis 4*, organizational career management practices are positively related to workers’ perceived employability, were *not accepted* for Study 1.

#### Structural Model for Study 2

**FIGURE 2:**
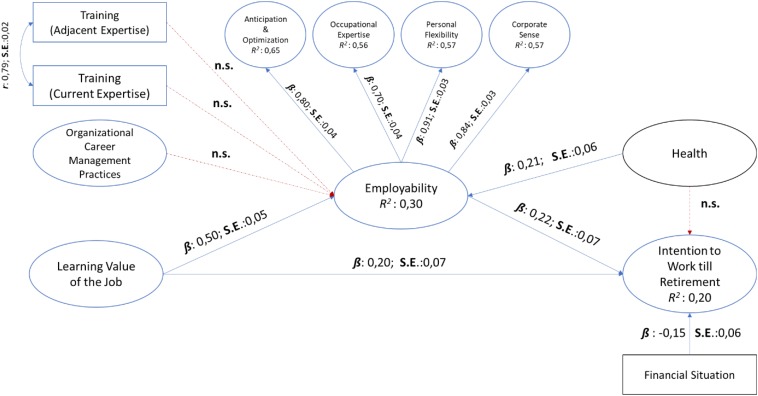
Structural model for Study 2 with control variables.

The structural model of the best-fitting measurement model (Model 1) for Study 2 showed acceptable fit *with the control variables* [*c.f.* Figure 2: χ^2^_(1809, *N* = 223)_ = 4732.07, *p* > 0.001; TLI = 0.96; CFI = 0.95; RMSEA = 0.07 [CI 95%:0.068,0.073]; SRMR = 0.07].

For the model with the control variables, the results showed that training and education (current expertise), training and education (adjacent expertise) and organizational career management practices did not produce statistically significant paths to perceived employability (*p* > 0.01). Learning value of the job related positively to perceived employability and declared 30% of the variance therein (β: 0.50; *SE*:0.05; *p* < 0.01). Further, perceived employability statistically significantly predicted 20% of the variance within intention to continue working until one’s retirement age (β: 0.22; *SE*:0.07; *p* < 0.01). In contrast to Study 1, learning value of the job directly related to intention to continue working until one’s retirement age (β: 0.20; *SE*: 0.07; *p* < 0.01); contributing to the 20% declared variance therein. Similar to Study 1, perceived health influenced perceived employability (β:0.21; *SE*:0.06; *p* < 0.01) but not intention to continue working until one’s retirement age (*p* > 0.01). Perceived financial situation again negatively influenced participants’ intention to continue working until their retirement age (β: −0.15; *SE*: 0.06; *p* < 0.01).

Therefore, *Hypothesis 1*, which stated that perceived employability is positively related to intention to continue working until one’s retirement age, and *Hypothesis 3*, which stated that the learning value of the job is positively related to perceived employability, is *accepted* for Study 2. However, *Hypotheses 2a and 2b*, training and education in current area of expertise (H2a) and in adjacent area of expertise (H2b) is positively related to workers’ perceived employability, and *Hypothesis 4*, organizational career management practices are positively related to workers’ perceived employability, were *not accepted* for Study 2.

### Robustness Check

Given the contemporary criticisms regarding the misuse of statistical control variables (*c.f.* Spector and Brannick, 2011), structural path models for both the controlled (i.e., controlling for perceived health and perceived financial situation) and uncontrolled conditions (i.e., model without perceived health and perceived financial situation) were computed and contrasted.

For Study 1, the structural model for the best-fitting measurement model (Model 1) showed acceptable fit *with* [χ^2^_(1809, *N* = 223)_ = 3855.00, *p* > 0.001; TLI = 0.93; CFI = 0.92; RMSEA = 0.07 [CI 95%:0.068,0.074]; SRMR = 0.07] and *without* [χ^2^_(1524, *N* = 223)_ = 3361.56, *p* > 0.001; TLI = 0.93; CFI = 0.93; RMSEA = 0.07 [CI 95%:0.070,0.077]; SRMR = 0.07] the control variables. The scale corrected S-B χ^2^ difference test showed that the model without the control variables fitted the data better than the model with the control variables (Model with vs. Model without: ΔS-B χ^2^ = 513.62; Δ*df* = 285; Scaling Correction: 0.66). Therefore, the model with the control variables was retained for the final analyses as both perceived health and perceived financial situation statistically significantly influenced the model fit. For Study 1, the model without the control variables produced similar results as the model with the control variables (*c.f.* Appendix A), however the strength of the relationship between perceived employability and intention to work until one’s retirement age was slightly stronger (β*:* 0.38; *SE*: 0.07; *p* < 0.01). Further the amount of variance of employability declared by learning value of the job (*R*^2^ = 0.25) was slightly lower. Similarly, the variance declared by perceived employability within intention to work until one’s retirement age was also slightly lower (*R*^2^ = 0.16).

For Study 2, both the models *with* [*c.f.* Figure 2: χ^2^_(1809, *N* = 223)_ = 4732.07, *p* > 0.001; TLI = 0.96; CFI = 0.95; RMSEA = 0.07 [CI 95%:0.068,0.073]; SRMR = 0.07] and *without* [χ^2^_(1524, *N* = 223)_ = 4096.02, *p* > 0.001; TLI = 0.96; CFI = 0.96; RMSEA = 0.07 [CI 95%:0.069,0.075]; SRMR = 0.06] the control variables showed acceptable fit. The scale corrected S-B χ^2^ difference test showed that the model without the control variables fitted the data better than the model with the control variables (Model with vs. Model without: ΔS-B χ^2^ = 655.88; Δ*df* = 285; Scaling Correction: 0.71). Therefore, the model with the control variables was retained as both perceived health and perceived financial situation influenced the model fit. For Study 2, the model without the control variables produced comparable results with to the model with the control variables (*c.f.* Appendix B). However, the strength of the relationship between perceived employability and intention to work until one’s retirement age (β: 0.24; *SE*:0.07; *p* < 0.01) was slightly higher. Further the amount of variance of perceived employability declared by learning value of the job (*R*^2^ = 0.26) and perceived employability within intention to continue working until one’s retirement age was also slightly lower (*R*^2^ = 0.17). Finally, the strength of the relationship between learning value of the job and intention to work until one’s retirement age was slightly lower between models (β*:* 0.24; *SE*:0.07; *p* < 0.01).

### Assessing the Indirect Effect of Perceived Employability

Based on the structural models for Study 1 and Study 2, the procedure proposed by Preacher et al. (2010) was used to determine the indirect effect of perceived employability on the relationship between learning value of the job and intention to work until one’s retirement age. The BCB method with 50,000 iterations was employed to construct a two-sided bias-corrected confidence interval at the 95% marker for each study. Given that no statistically significant relationships existed between training and education as well as organizational career management practices and perceived employability, the two associated indirect effect *Hypothesis* (*5a* and *5b*) were *automatically not accepted*.

Table 5 provides an overview of the results. It shows that perceived employability indirectly affects the relationship between learning value of the job and intention to work until one’s retirement age in both samples. The range of the estimate through the BC CI does not include zero, and therefore there is evidence that perceived employability plays a mediating role in the aforementioned relationship. Therefore, *Hypothesis 5c*, which stated that perceived employability indirectly affects the relationship between the learning value of the job and intention to continue working until one’s retirement age, is *accepted* for both Study 1 and 2.

**TABLE 5:** Indirect effects of perceived employability on the relationship between learning value of the job and intention to work until one’s retirement age.

**Variable**	**Estimate**	***SE***	**95% BC CI**
Study 1: Indirect effect	0.11	0.06	[0.14; 0.30]
Study 2: Indirect effect	0.11	0.05	[0.03; 0.22]

*SE, standard error; BC CI, bias-corrected confidence interval.*

## Discussion

### Reflections Upon the Results

The present study aimed to shed light on the question what enables and motivates older workers to continue working until their official retirement age. To reach this aim, we examined (a) the relationship between perceived employability and the intention to continue working until one’s retirement age, and (b) the relationships of potential antecedent factors at three different levels with perceived employability using two different samples of older (45-plus) white collar workers.

In total, three out of our five hypotheses were (partly) confirmed, and the results for both samples were similar, which contributes to the robustness of our findings. Firstly, our results showed that a higher level of perceived employability is indeed related to a stronger intention of older (45-plus) workers to continue working until one’s official retirement age. So, investing in the employability of this specific group seems to pay off. However, based on the results of our study, not all types of investments seem to contribute equally to a higher level of (perceived) employability. In both samples it was found that investing in the learning value of the job is the most promising strategy, which is in line with JD-R theory (Demerouti et al., 2001; Schaufeli and Bakker, 2004) and with the results of earlier research (Van der Heijden, 2001). So, designing a challenging work environment that offers chances and opportunities for growth and development is not only important for younger workers, but also for older ones (cf. De Lange et al., 2008). This corroborates the results of the study by Van Dam et al. (2009) who found that employees might postpone early retirement when they anticipate to work in a challenging and rewarding work environment. Moreover, Van Solinge and Henkens (2008) also found that learning/growth opportunities in one’s job were significantly positively related to workers’ actual retirement age. Given the demographical trends that were mentioned in the introduction section, the importance of policies and practices that stimulate (prolonged) labor participation of older workers can only be expected to increase.

In the current study, neither training and education nor organizational career management practices contributed significantly to employees’ self-perceptions of employability. This is not in line with JD-R theory (Demerouti et al., 2001; Schaufeli and Bakker, 2004) and with results of earlier studies that did find significant relationships between these types of human capital/HR-investments and employability (amongst others, Forrier and Sels, 2003; Berntson et al., 2006; Verbruggen et al., 2008; Van Vuuren et al., 2011). Possibly, it is in particular the (practical) applicability of training and education when someone is ‘back at the workplace’ that enhances one’s employability (see also Van der Heijden et al., 2016) instead of the mere participation in this type of activities.

A possible explanation for the outcomes regarding the organizational level antecedent, i.e., career management practices, may be that in the current study we asked employees about the extent to which their organization offers support in career management. However, despite of the fact that organizations offer this type of support, employees may decide to not or hardly make use of it thereby not significantly affecting their employability. A similar process occurs in the area of promoting work-life balance in organizations. Here we see that offering work-family policies is useless as long as the organizational culture is not family supportive (Kinnunen et al., 2014). In addition, lately there has been a shift in many organizations where the HR department focuses more on strategic HR responsibilities while the responsibility of operational HR tasks is transferred to line management (Bos-Nehles et al., 2013). Intended HR practices are designed and developed on a strategic level by the HR department (Paauwe and Boselie, 2005), and responsibilities of HR specialists for daily HR tasks are handed over to line managers (so-called devolution; Bos-Nehles et al., 2013). Depending on the HR competences of line managers, differences may exist between how HR practices are intended and how they are eventually implemented (Wright and Nishii, 2007). When implemented practices differ from how they were intended by HR management, this may affect employees’ perceptions of these practices, which in turn impacts their (re)actions in response to these practices.

Our results also showed that people who feel healthier consider themselves as more employable. This is in line with the point of view of Tuomi et al. (2001) who state that workers’ health is the defining element of their workability. In contrast to Wöhrmann et al. (2017) who found that the relationship between respectful leadership and older workers’ desired retirement age was mediated by subjective health, we did not find a relationship between workers’ perceived health and their intention to continue working until their retirement age. However, we did find that a perceived good financial situation is a push factor to retire early, or at least not to continue working until one’s official retirement age. This finding corroborates the results of earlier studies by Talaga and Beehr (1989), Zappala et al. (2008), and Fasbender et al. (2016), and again, points at the importance of designing jobs that are intrinsically motivating (i.e., have a high learning value) to retain older workers in the labor market – especially if they don’t have a need to continue working for financial reasons.

Altogether, our results contribute to contemporary theorizing on retirement research in that they shift the (traditional) focus from the reasons for retiring early to reasons to continue working until one’s official retirement age. By doing so this study aligns with the current positive approach in psychology (Seligman and Csikszentmihalyi, 2000) focusing on positive human functioning (= continuing to work until one’s retirement age) instead of on preventing maladaptive and more negative behavior (= retiring early). In addition, our study uncovers perceived employability as a working mechanism underlying the relationship between specific work-related factors and the intention to continue working until one’s official retirement age. This is important because it helps us to better understand why certain measures are effective when it comes to raising the average retirement age whereas others are not. Moreover, in contrast to many earlier studies that focused on antecedents of employability at a single level only, our study included antecedents at three different levels simultaneously. As such, we adopted an interactionist perspective (Lewin, 1935; Endler and Magnusson, 1976) and responded to earlier calls for a more integrative approach, which is also stressed in the current thinking on sustainable careers. In particular, building upon the theoretical framework by De Vos et al. (2018), a systemic approach, using a multiple-stakeholder perspective (cf. Colakoglu et al., 2006) to understand the possible impact of different parties involved [such as individual employees and the (HR) management of organizations], has been used.

### Limitations and Suggestions for Future Research

The findings of this study should be interpreted in the light of several limitations. First, all data were collected at one point in time, so no causal inferences can be made based on our results. A related problem with a cross-sectional design is that it makes it difficult to study retirement as a process that unfolds over time. In order to study these processes in more detail, future research should try to incorporate longitudinal designs which are also more in line with a lifespan development perspective on (late) career planning (e.g., Fasbender et al., 2019). In addition, future studies should also consider the possibility of gradual retirement and examine the factors that determine the motivation to retire either fully or gradually. Gradual retirement is an umbrella term for phased and partial retirement. Phased retirement is staying in the original job but working fewer hours. Partial retirement refers to the transition to a less demanding bridge job or to self-employment. Gradual employment enjoys high popularity in the United States and is becoming more frequent in Europe too (Bloemen et al., 2016).

Although it has been argued that self-report data are valid when examining perceptual outcomes (Chan, 2009), it would be desirable for future research to collect data from other sources as well, e.g., supervisors’ or colleagues’ perceptions of one’s employability and/or functioning at work. In terms of measurement issues, it is advisable to look for new ways of measuring the two training variables because people might not always have an accurate assessment of the amount of days they spend on training over a year. In this respect it would be interesting if HR data on the training of personnel could be made available for research.

Thirdly, despite of the fact that we found similar results in two different samples, both of these samples consisted of white collar workers from the same country. Future research should test our research model and related hypotheses in different samples of e.g., blue collar workers, and workers from other occupational sectors and/or from other countries too. For example, in samples of blue collar workers in physically demanding jobs, perceived health could be significantly related to the intention to continue working until one’s retirement age whereas we did not find this relationship in our current, white collar samples. Moreover, in this study workers were classified as ‘older’ based on their calendar age. However, recent studies have demonstrated that other types of age categorizations are relevant in relation to employability too (Sterns and Miklos, 1995; Le Blanc et al., 2017). In addition, individuals are not independent actors in the transition to retirement. They are embedded in social relationships (e.g., partner, family, friends, co-workers) and active in different life spheres that function interdependently, such that experiences in one life sphere can influence other spheres (Fisher et al., 2016). Future research would benefit from integrating family life sphere factors in current research model.

Another point of attention in the retirement debate refers to the relationship between working conditions and (early) retirement. A recent review suggests that high-quality psychosocial working conditions may encourage later retirement (Browne et al., 2019). On the opposite, for those who are employed in jobs characterized by bad workings conditions (such as a high level of physical demands) there appears to be an immediate positive effect of retirement on health (Mazzonna and Peracchi, 2017). Efforts to extend working life should therefore also address issues relating to the immediate psychosocial work environment.

Finally, future research is needed to establish the predictive validity of our research model for actual retirement. Further empirical work adding some other valuable predictors and/or moderators to the currently used model could deepen our understanding of the relationship between employability and retirement (intentions). One promising factor in this respect could be the employability culture of an organization. In such a culture, individual development and employability are fostered. Research by Nauta et al. (2009) showed a positive relationship between employability culture and employability orientation and a negative relation between employability culture and turnover intentions. This suggests that such a culture stimulates employees to engage in task changes and development opportunities, which decreases employees’ intentions to leave their job. Parallel to this finding, one could speculate that employees who perceive a good employability culture are more prone to continue working until their official retirement age.

### Practical Implications

First, our scholarly contribution suggests that informal learning potential in the job itself is an important factor in the light of fostering one’s employability. This is in line with the results of a study by Fasbender et al. (2014) that showed that organizations that want their employees to engage in *post-retirement* employment, i.e., to continue working after their official retirement age, should design the work for older workers to enable development. In their framework of the development of knowledge and skills at work, Eraut et al. (2000) classify informal learning as a key process. Most informal learning (in contrast to formal learning or training) takes place through learning from other people or by learning from experience (Eraut, 2004). One way of stimulating informal learning is by engaging in job crafting behaviors. Job crafting is defined as the changes individuals make in their task or relational boundaries, as well as cognitive changes in perceptions of their work, in order to find more meaning in their job (Wrzesniewski and Dutton, 2001) or to make the job more fitting with individual preferences and needs (Tims and Bakker, 2010). Since research has shown that job crafting can have significant positive effects on employee well-being, motivation and performance (see Demerouti, 2014, for an overview), interest in ways of stimulating job crafting has also grown significantly. Recently, Demerouti et al. (2019) reviewed studies on the effectiveness of a theory- based intervention to stimulate job crafting behaviors and concluded that the intervention appeared effective in improving forms of proactive behavior at work, which can be considered an example of informal learning. Thus, the employability, and in turn also the retention of (older) people in our ‘shrinking’ workforce can be safeguarded. This is particularly important for future generations of older workers, as recent research has shown that Millenials are most likely to be attracted to workplaces that provide, amongst others, interesting work and that their work values are relatively stable as they grow older and gain work experience (Kuron et al., 2015).

## Data Availability

The datasets generated for this study are available on request to the corresponding author.

## Ethics Statement

Ethical review and approval was not required for the study on human participants in accordance with the local legislation and institutional requirements. The ethics committee waived the requirement of written informed consent for participation.

## Author Contributions

MP was responsible for the conception and design of the study and organized the database. PL performed the original statistical analyses and wrote the first draft of the manuscript. MP and BH wrote sections of the manuscript. LZ performed and wrote up the statistical analyses for the revision. All authors contributed to manuscript revision, read and approved the final submitted version.

## Conflict of Interest Statement

The authors declare that the research was conducted in the absence of any commercial or financial relationships that could be construed as a potential conflict of interest.

## Figures and Tables

**APPENDIX A:**
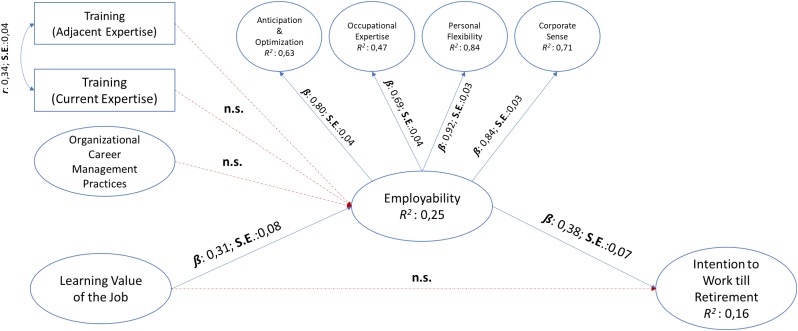
Structural model for Study 1 *without* control variables.

**APPENDIX B:**
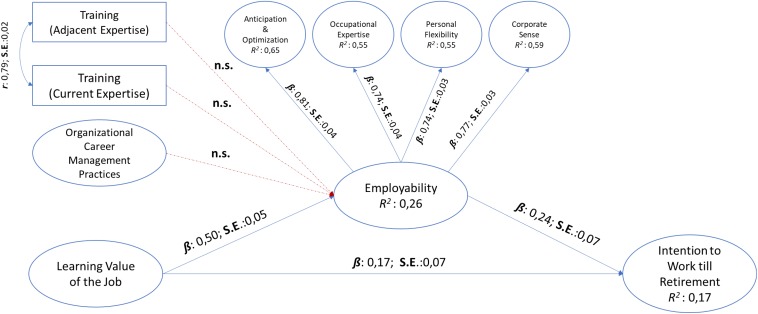
Structural model for Study 2 *without* control variables.
